# The Role and Mechanism of Transglutaminase 2 in Regulating Hippocampal Neurogenesis after Traumatic Brain Injury

**DOI:** 10.3390/cells12040558

**Published:** 2023-02-09

**Authors:** Ruo-Xi Shi, Cong Liu, Ya-Jie Xu, Ying-Ying Wang, Bao-Dong He, Xuan-Cheng He, Hong-Zhen Du, Baoyang Hu, Jianwei Jiao, Chang-Mei Liu, Zhao-Qian Teng

**Affiliations:** 1 State Key Laboratory of Stem Cell and Reproductive Biology, Institute of Zoology, Chinese Academy of Sciences, Beijing 100101, China; 2 Savaid Medical School, University of Chinese Academy of Sciences, Beijing 100408, China; 3 Beijing Institute for Stem Cell and Regenerative Medicine, Beijing 100101, China; 4 Institute for Stem Cell and Regeneration, Chinese Academy of Sciences, Beijing 100101, China

**Keywords:** traumatic brain injury, transglutaminase 2, neuronal stem/progenitor cell, neurogenesis, hippocampus

## Abstract

Traumatic brain injury usually results in neuronal loss and cognitive deficits. Promoting endogenous neurogenesis has been considered as a viable treatment option to improve functional recovery after TBI. However, neural stem/progenitor cells (NSPCs) in neurogenic regions are often unable to migrate and differentiate into mature neurons at the injury site. Transglutaminase 2 (TGM2) has been identified as a crucial component of neurogenic niche, and significantly dysregulated after TBI. Therefore, we speculate that TGM2 may play an important role in neurogenesis after TBI, and strategies targeting TGM2 to promote endogenous neural regeneration may be applied in TBI therapy. Using a tamoxifen-induced *Tgm2* conditional knockout mouse line and a mouse model of stab wound injury, we investigated the role and mechanism of TGM2 in regulating hippocampal neurogenesis after TBI. We found that *Tgm2* was highly expressed in adult NSPCs and up-regulated after TBI. Conditional deletion of *Tgm2* resulted in the impaired proliferation and differentiation of NSPCs, while *Tgm2* overexpression enhanced the abilities of self-renewal, proliferation, differentiation, and migration of NSPCs after TBI. Importantly, injection of lentivirus overexpressing TGM2 significantly promoted hippocampal neurogenesis after TBI. Therefore, TGM2 is a key regulator of hippocampal neurogenesis and a pivotal therapeutic target for intervention following TBI.

## 1. Introduction

Traumatic brain injury, a form of brain damage caused by external mechanical forces [1], is a major health and socioeconomic issue worldwide [2,3]. Millions of survivors of TBI tend to have mental, physical, or intellectual disabilities [4]. TBI manifests in time-dependent ways and unfolds in phases following the traumatic event [5]. Primary brain damage is followed by a series of secondary molecular and cellular events that can result in cell death, tissue atrophy, and neurodegeneration over time [6,7]. Due to the heterogeneity and complexity of TBI, there is still no effective rehabilitation treatment for TBI [8]. Most clinical interventions focus on minimizing secondary injury, but neural repair and regeneration are very limited [1,9].

During the past few decades, endogenous neural stem/progenitor cells (NSPCs) have opened a new therapeutic avenue for brain injuries [10,11,12,13]. Unfortunately, endogenous NSPCs lose their abilities to migrate and differentiate into mature neurons at the injury sites in adult brain [14,15,16,17]. Therefore, developing new strategies to promote neural differentiation of endogenous NSPCs is crucial for replacing neurons lost in TBI.

Transglutaminases are a family of enzymes that form calcium-dependent intermolecular isopeptide bonds between glutamine and lysine [18]. Transglutaminase 2 (TGM2) is ubiquitously expressed in the central nervous system and regulates numerous cellular processes, including calcium-dependent intracellular signaling, neuroinflammation, protein cross-linking, and cell death [18,19,20,21]. Overexpression of TGM2 in transplanted ectomesenchymal stem cells increases their differentiation into neuron-like cells in adult rats with spinal cord injury [22,23]. Notably, upregulation of TGM2 is observed in cortical and hippocampal tissues of adult rats that received a controlled cortical impact injury [24]. Interestingly, TGM2 is highly expressed in NSPCs and has been identified as a crucial component of the neurogenic niche [25]. Retinoic acid treatment results in increased TGM2 expression in human neuroblastoma SH-SY5Y cells, and TGM2 may contribute to neuronal differentiation through the cAMP-CREB pathway [26]. However, the role and mechanism of TGM2 in regulating neurogenesis following TBI are still unclear.

In this study, we demonstrate that TGM2 plays an important role in regulating NSPCs proliferation and differentiation. Lentiviral overexpression of TGM2 significantly promotes hippocampal neurogenesis after TBI, suggesting TGM2 is a potential target of stem cell therapy for TBI.

## 2. Materials and Methods

### 2.1. Mice

Floxed *Tgm2* mice (*Tgm2^fl/fl^*) were generated by Cyagen Biosciences (Suchow, China) using CRISPR/Cas9-mediated genome editing. The distinct locations of the *Tgm2* introns 3 and 4 were targeted by two sgRNAs (forward: GACGTATAAACCTCACCGCAAGG; reverse: ATTCCCCGTGACTAGTGCGGGGG). *Nestin-cre*ER^T2^ mice (Jax Stock No. 016261) were crossed with *Tgm2^fl/fl^* mice to produce tamoxifen-inducible knockout mice of *Tgm2* in NSPCs (*Tgm2^fl/fl^*; *Nestin-cre*ER^T2^, hereafter referred to as *Tgm2*-iKO). *Tgm2^fl/fl^* littermates were used as controls (*Tgm2*-WT). The mice were socially housed (2–5 mice/cage) and given ad libitum access to food and water during a 12-h light-dark cycle. Investigators were blinded to genotype of mice for all experiments. All animal experiments were approved by the Animal Committee of the Institute of Zoology, Chinese Academy of Sciences.

### 2.2. Blade Penetrating Stab Wound to the Hippocampus

To produce a homogeneous hippocampal injury, male mice at 6–8 weeks of age were anesthetized with tribromoethanol (375 mg/kg) and received a blade penetrating stab wound injury in the right hippocampus according to published protocols [27,28]. Briefly, the scalp was disinfected and cut along the midline to expose the skull. A cranial window (4 mm in length, 1 mm in width) was opened on the right side of the skull, and a sterile scalpel blade #15 was inserted vertically into the hippocampus with the following coordinates (from bregma): AP (anterior-posterior) = 1.4 mm, ML (mediolateral) = 1.4 mm; AP = 4.3 mm, ML = 2.4 mm; DV (dorsal-ventral) = 3.5 mm. After surgery, a povidone-iodine solution was applied to prevent infections.

### 2.3. Tamoxifen Induction and BrdU Labeling

Tamoxifen (Sigma-Aldrich, St. Louis, MO, USA, T5648) was dissolved in corn oil (Sigma-Aldrich, C8267). Adult mice (6–8 weeks old) received three intraperitoneal injections of tamoxifen to initiate genetic recombination at a dose of 180 mg/kg, each injection was separated by 24 h. For BrdU labeling, mice received daily intraperitoneal BrdU (Sigma-Aldrich, B5002-5G) injections after TBI or sham surgery at a dose of 100 mg/kg for three consecutive days.

### 2.4. Cell Cultures and Collection of Conditioned Medium

HEK293 cells were cultured in DMEM medium (Gibco, Carlsbad, CA, USA C11995500BT) containing 10% FBS (Gibco, A4766801) and 1% penicillin-streptomycin (Hyclone, Logan, UT, USA, SV30010) at 37 °C in a humidified incubator with 5% CO_2_.

Primary NSPCs were isolated from the cerebral cortex of E13.5 mice and cultured according to our previous method [29]. In brief, the cerebral cortex was dissociated with TrypLE Express (Gibco, 12604013) for 8 min. DMEM/F-12 medium (Gibco, 10565-018) containing 10% FBS, 2 mM L-glutamine (Gibco, 25030-0-048) and 1% penicillin-streptomycin solution were added to stop digestion. NSPCs were then collected and cultured in proliferation medium, DMEM/F-12 supplemented with 20 ng/mL FGF (PeproTech, Rocky Hill, NJ, USA, K1606), 20 ng/mL EGF (PeproTech, A2306), 0.5% N2 supplement (Gibco, A13700701), 1% B27 supplement (Gibco, 17504044), and 1% penicillin–streptomycin. Half of the cultured medium was replaced with fresh medium every two days. For RNA sequencing, 1 μM of (Z)-4-hydroxytamoxifen (Sigma-Aldrich, H7904-5MG) was added in the culture medium for 8 days to induce TGM2 deletion. Cells were then cultured under either proliferating or differentiating conditions for 48 h to analyze the transcriptional profile changes triggered by TGM2 deletion. The differentiation medium was DMEM/F-12 containing 1% N2 supplement, 1% penicillin–streptomycin, 1 μM retinoic acid (Sigma-Aldrich, R-2625), and 5 μM forskolin (Sigma-Aldrich, F-6886).

Microglia was cultured as described previously [30]. Briefly, P3 cortices were dissociated and digested at 37 °C for 10 min with TrypLE Express. After that, DMEM/F12 medium supplemented with 10% FBS was used to culture mixed glial cells for 2 weeks. Microglia were then purified by shaking flasks for 2 h at 130 rpm. For collecting microglial conditioned medium, primary microglia were treated with 100 ng/mL of LPS (Sigma-Aldrich, L2630) for 24 h. Medium were collected and centrifuged for 5 min at 500 rcf. Finally, the supernatants were aliquoted and stored at 80 °C until use.

### 2.5. Immunostaining

Immunohistochemical staining (IHC) was performed as described previously [29]. After fixation in 4% paraformaldehyde (PFA) overnight and dehydration with 30% sucrose solution, brains were cut into 35 μm thick sections. For immunocytochemical staining (ICC), glass coverslips coated with poly-L- ornithine/laminin (PLL) were used to seed primary cells. The brain sections or cells on coverslips were post-fixed in 4% PFA for 20 min before immunostaining, then blocked in 2% BSA (Easybio, Beijing, China, BE6254) containing 0.3% Triton (Sigma-Aldrich, T8787) for 2 h. Samples were incubated with primary antibodies in a blocking solution overnight at 4 °C. After washing with PBS for 30 min, samples were then incubated with secondary antibodies for 2 h at room temperature. Finally, samples were mounted on glass slides with antifade adhesive medium. The primary antibodies included anti-Tgm2 (1:500, Abcam, Cambridge, MA, USA, ab2386), anti-Nestin (1:1000, Cell signaling, Danvers, MA, USA, 4760T), anti-BrdU (1:500, Abcam, ab6326), anti-Tuj1 (1:1000, Biolegend, San Diego, CA, USA, 801202), anti-GFAP (1:500, Abcam, 16825-1-AP), anti-Doublecortin (1:500, Abcam, 4604s) and anti-NeuN (1:1000, Millipore, Billerica, MA, USA, MAB377) anti-SOX2(1:500, Santa, Santa Cruz, CA, USA, sc-17320). Alexa Fluor conjugated secondary antibodies were purchased from Life Technologies (Gaithersburg, MD, USA) and used at the concentration of 1:500 dilution. Nuclei were counterstained with DAPI (1:1000; Sigma-Aldrich, D9542).

For co-localization analysis of marked proteins in cells, z-stack images were taken with a LSM880 confocal microscope (Zeiss, Oberkochen, Gernany). Co-localization was determined as overlap with orthogonal view from different planes (x/y, x/z, and y/z). For quantifying the fluorescence intensity, images from all groups were captured at the same exposure time. The mean fluorescence of small areas that had no positive signals was measured as the background reading for every image. The corrected total square fluorescence was calculated with the following equation: the Corrected Total Frame Fluorescence = Integrated Density − (Mean Fluorescence of Background readings × Area of Selected Frame).

### 2.6. Quantitative RT-PCR

Total RNA from cells or tissues was extracted with TRIzol reagent (Invitrogen, Carlsbad, CA, USA, 15596018). The Transcript One-Step gDNA Removal and cDNA Synthesis Kit (TransGen Biotech, Beijing, China, 11104ES70) was used to reverse-transcribed RNA into cDNA. For each sample, quantitative RT-PCR (qRT-PCR) reactions were run in triplicate in a 20 μL system with the SYBR Green Master Mix (Yeasen Biotech, Shanghai, China, 11201ES08). β-actin was used as an endogenous control. The 2^−ΔΔCT^ method was used to measure relative expression levels of genes. PCR reactions were run at 95 °C for 30 s, followed by 45 cycles of denaturation at 94 °C for 10 s, annealing at 60 °C for 30 s, and elongation at 72 °C for 30 s. All qRT-PCR experiments were repeated at least three times. The primers for qRT-PCR are listed in Table 1.

### 2.7. Western Blot

Total protein was extracted with RIPA buffer (Beyotime, Shanghai, China, P0013B) supplemented with 10 mM PMSF (Beyotime, ST505). Protein concentrations were calculated by the BCA protein assay kit (Thermo Fisher, Waltham, MA, USA, A101-01). Protein samples in equal amounts were loaded onto a 10% SDS-PAGE gel. After electrophoresis, proteins were transferred to a PVDF membrane, blocked with 5% skim milk, and incubated with primary antibodies overnight at 4 °C, including anti-β-Actin (1:5000, Easybio, BE0033-100) and anti-Tgm2 (1:2000, Abcam, ab2386). Then membranes were washed in TBST and incubated with the secondary antibodies at room temperature for 2 h. Finally, the immunoreactive bands were detected with the chemiluminescence reagent (ECL, 34580).

### 2.8. Lentivirus Production and In Vivo Grafting

Lentiviral vectors were constructed and packaged as described previously [31,32]. For overexpressing *Tgm2*, *Tgm2* cDNA was amplified from mouse cerebral cortex by PCR, and then subcloned into the construction of pCD511B-copGFP vector containing CMV promoter. For knocking down *Tgm2*, a U6 promoter-driven *Tgm2* shRNA (5′-CCAAGTATGATGCACCCTT-3′) hairpin loop sequence was inserted into the lentiviral construct.

To produce lentivirus, HEK293T cells were co-transfected with lentiviral vector and packaging plasmids (pMDLg/pRRE, pRSV-Rev, and pMD2.VSV-G) using polyethylenimine. Lentiviral supernatants were collected at 48 h and 72 h after transfection and filtered through a 0.22 μm filter, then centrifuged at 19 k rpm for 2 h at 4 °C. After dissolving in PBS, lentivirus with a titer of 1 × 10^9^ TU/mL was aliquoted and stored at −80 °C until use.

Lentivirus was stereotaxically grafted into the dentate gyrus (DG) of 6–8 week-old *Nestin-cre*ER^T2^;tdTamato male mice using the coordinates relative to bregma as follows: anteroposterior, −1.2/2.0 mm; lateral, ±2.0/2.8 mm; ventral, −1.6/1.9 mm (from dura). For each animal, 1 μL of the control lentivirus was injected into the left DG, and 1 μL of the *Tgm2*-OE or sh*Tgm2* lentivirus was injected into the right DG.

### 2.9. Proliferation, Differentiation, Self-Renewal, and Migration Analyses of Cultured NSPCs

Analyses of proliferation, differentiation, self-renewal, and migration were performed as previously described [32,33]. To examine the proliferation ability of NSPCs, cells were plated on poly-L-ornithine/laminin (PLL)-coated coverslips at a density of 5 × 10^4^ cells per well. Lentivirus was added into the proliferation medium at 6 h post-plating. Following lentiviral transduction for 48 h, 5 μM BrdU was added into the culture medium for 6 h. NSPCs were fixed with 4% PFA for 20 min at room temperature, and then washed with PBS and immunostained with anti-BrdU.

To examine the self-renewal ability, we plated NSPCs in uncoated 12-well plates at a density of 5 × 10^4^ cells/well. Following lentiviral transduction for 48 h, the sizes of primary and secondary neurospheres were measured.

For the differentiation assay, NSPCs were seeded on PLL-coated coverslips at a density of 1 × 10^5^ cells/well in 24-well plates. Following lentiviral transduction for 48 h, NSPCs were cultured in differentiation medium, DMEM/F-12 containing 1% N2 supplement, 1% penicillin–streptomycin, 1 μM retinoic acid, and 5 μM forskolin, for 3 days. After fixing cells with 4% PFA for 20 min at room temperature and washing with PBS, NSPCs were immunostained with anti-Tuj1 and anti-GFAP antibodies.

Scratch wound healing assay was carried out to evaluate the migration capacity of NSPCs. NSPCs were seeded on PLL-coated coverslips at a density of 1 × 10^5^ cells/well in 24-well plates, and treated with lentiviral transduction for at least 48 h. After cell confluency reached more than 90%, scratch wounds were generated using a 200 μL micropipette tip. Cells were then allowed to grow for additional 48 h, and the closure of wound was photographed at 0 h and 48 h, respectively.

### 2.10. RNA Sequencing

Total RNA was extracted from the primary cultured E13.5 NSPCs using TRIzol reagent. Libraries were constructed and sequenced on Illumina 2500 HiSeq platforms at Annoroad Gene Technology (Beijing, China) Data quality was assessed using FastQC, and adapter sequencing was removed with FASTX-Toolkit. Salmon (v1.0.0, SAF Pattern) was used for alignment-based quantification of mappings. Through GENCODE vM23, clean reads were mapped to the reference genome of the mouse. DESeq2 was used to identify differences in gene expression across all samples. Differentially expressed genes were determined with *p* < 0.05, and log2FC > 1.5 or < −1.5. Gene Ontology (GO) and Kyoto Encyclopedia of Genes and Genomes (KEGG) pathway enrichment analysis were subsequently performed. As described previously, a pseudoreplication analysis of all samples was conducted to reduce the influence from libraries construction bias and sequencing depth [34]. The RNA-seq data have been deposited in the Genome Sequence Archive in the National Genomics Data Center, Beijing Institute of Genomics, Chinese Academy of Sciences with accession number CRA009304.

### 2.11. Statistical Analysis

To determine statistical significance, either unpaired two-tailed *t*-test (to compare the mean of two independent groups) or two-way ANOVA test (to determine the effect of two variables on an outcome) were performed using GraphPad Prism v8.01. All data are shown as mean ± SEM (standard error of the mean). *p*-values less than 0.05 were considered as statistically significant.

## 3. Results

### 3.1. Tgm2 Is Upregulated in NSPCs following TBI

**Figure 1:**
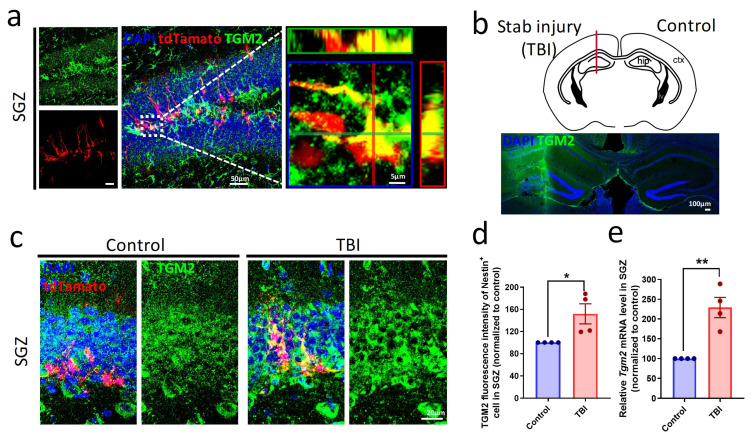
TGM2 is expressed in hippocampal NSPCs and upregulated after TBI. (**a**) Representative images of TGM2 immunostaining in the subgranular zone (SGZ) of *Nestin-cre*ER^T2^;tdTamato mice. The regions within the dotted white boxes are shown in a higher magnification 3D view (right panel). Scale bars: 50 µm (left and middle panels), 5 µm (right panel). (**b**) Schematic (upper panel) and representative image (lower panel) of *Nestin-cre*ER^T2^;tdTamato mice at 7 days post-injury (dpi). Scale bar: 100 µm. (**c**,**d**) Representative images (**c**) and quantification (**d**) of TGM2 immunostaining in SGZ at 7dpi. Scale bar: 20 μm. (**e**) Quantification of *Tgm2* mRNA levels in SGZ at 7 dpi by real-time qPCR. *n =* 4 mice per group. Data are represented as means ± SEM; two-tailed *t*-test, * *p* < 0.05, ** *p* < 0.01.

To examine whether *Tgm2* is expressed in hippocampal NSPCs, we performed immunofluorescent staining of TGM2 in hippocampal tissues of 1-month-old *Nestin-cre*ER^T2^;tdTamato mice which had tamoxifen-induced tdTamato labeling of nestin-expressing stem cells and their progeny in neurogenic regions. We found that TGM2 was highly expressed in NSPCs (tdTamato^+^) in the subgranular zone (SGZ) of the hippocampus (Figure 1a). Using a mouse model of hippocampal stab wound injury (Figure 1b), we observed that both *Tgm2* mRNA and protein expression levels were significantly elevated in SGZ at 7 days after injury (Figure 1c–e), suggesting that TGM2 might play a pivotal role in neurogenesis following TBI.

### 3.2. Deletion of TGM2 in NSPCs Inhibits Hippocampal Neurogenesis

To explore the function of TGM2 in NSPCs, we generated the NSPCs-specific TAM-inducible *Tgm2* knockout (iKO) mice by crossing *Nestin-cre*ER^T2^ mice with *Tgm2^fl/fl^* mice (Figure S1a). Upon TAM administration to 6–8 weeks-old mice for three consecutive days, our qRT-PCR analysis demonstrated that the mRNA level of *Tgm2* was significantly reduced in the dentate gyrus of *Tgm2* iKO mice compared to that of WT mice (Figure S1b). Consistently, TGM2 fluorescence signal was almost undetectable in *Tgm2*-iKO NSPCs in SGZ by immunohistochemistry staining assays (Figure S1c–f). These results indicated that *Tgm2* could be specifically deleted in NSPCs upon TAM treatment.

**Figure 2:**
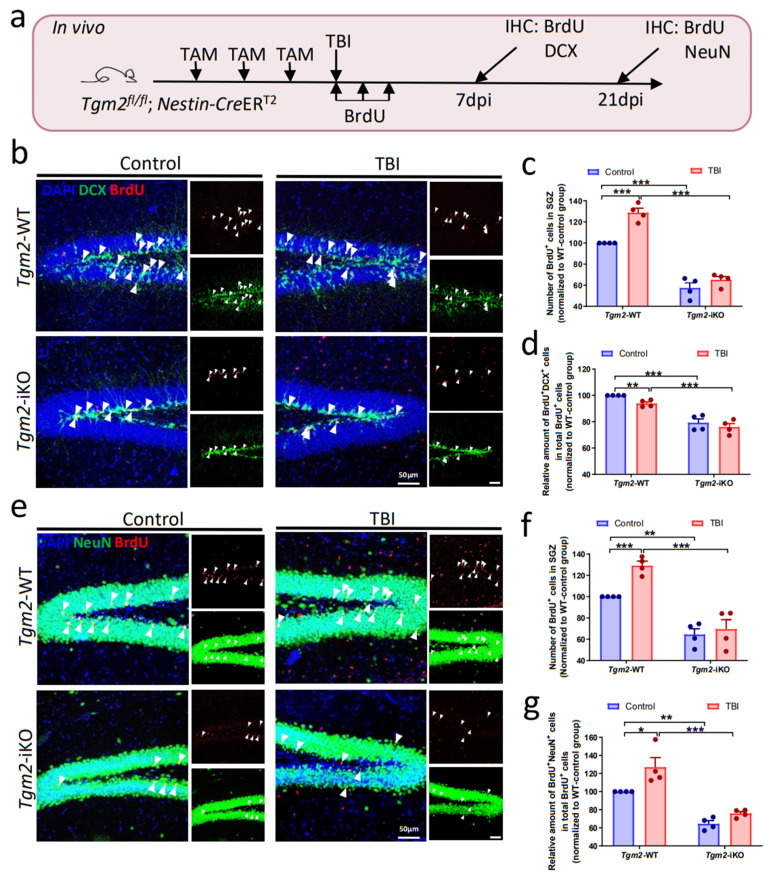
Deletion of TGM2 in NSPCs inhibits hippocampal neurogenesis after TBI in vivo. (**a**) Schematic illustration for analyzing neurogenesis in vivo. (**b**–**d**) Representative images (**b**) and quantification (**c**,**d**) of BrdU (red) and DCX (green) immunostainings of dentate gurus of *Tgm2* iKO and WT littermates at 7 dpi. Arrowheads indicate BrdU^+^DCX^+^ cells. Scale bar: 50 µm. (**e**–**g**) Representative images (**e**) and quantification (**f**,**g**) of BrdU^+^ cells (**f**) and BrdU^+^NeuN^+^ cells (**g**) in dentate gurus of *Tgm2* iKO and WT littermates at 21 dpi. *n =* 4 mice per group. Data are represented as means ± SEM; two-way ANOVA, * *p* < 0.05, ** *p* < 0.01, *** *p* < 0.001.

Next, we applied a unilateral hippocampal stab wound injury to 6–8 weeks-old *Tgm2* WT and iKO mice to examine whether TGM2 depletion affects proliferation and differentiation of NSPCs after TBI. Following intraperitoneal administration of TAM for three consecutive days, animals were subjected to TBI and administered BrdU (200 mg/kg body weight) daily for three consecutive days (Figure 2a). At 7 days post-injury (dpi), TBI treatment increased the number of BrdU^+^ cells (*F*_(1,12)_ = 26.91, *p* < 0.001), while TGM2-depletion significantly inhibited proliferation of NSPCs in the dentate gyrus (DG) (*F*_(1,12)_ = 227.2, *p* < 0.001) (Figure 2b,c). Meanwhile, both TBI treatment (*F*_(1,12)_ = 4.952, *p* < 0.05) and TGM2-depletion (*F*_(1,12)_ = 85.59, *p* < 0.001) resulted in reduced percentages of DCX^+^BrdU^+^ cells in total BrdU^+^ cells in DG, indicating that knockout of *Tgm2* represses neuronal differentiation of NSPCs following TBI (Figure 2d).

At 21 dpi, although TBI treatment promoted the production of both BrdU^+^ cells (*F*_(1,12)_ = 9.306, *p* < 0.05) and newborn neurons (NeuN^+^BrdU^+^) (*F*_(1,12)_ = 11.86, *p* < 0.01), TGM2 loss-of-function significantly reduced the amount of BrdU^+^ cells (*F*_(1,12)_ = 71.98, *p* < 0.001) and newborn neurons (*F*_(1,12)_ = 60.32, *p* < 0.001) in DG after injury (Figure 2e–g). By performing qRT-PCR analysis, we detected lower mRNA levels of *Gfap* and *Dcx* in the dentate gyrus of *Tgm2* iKO mice under both physiological and injured conditions compared to that of WT littermates (Figure S2d,e). Again, these results suggested that TGM2 is required for hippocampal neurogenesis following TBI.

### 3.3. Overexpression of TGM2 in NSPCs Promotes Neurogenesis In Vitro

To further explore the role of TGM2 in neurogenesis, we constructed specific lentiviral vectors with GFP expression to up- or down-regulate TGM2. Transduction of TGM2 overexpression (lenti-*Tgm2*-OE) and downregulation (lenti-sh*Tgm2*) lentiviral vectors in cultured E13.5 NSPCs led to robust upregulation and reduction of TGM2 expression, respectively (Figure S3a–d).

**Figure 3:**
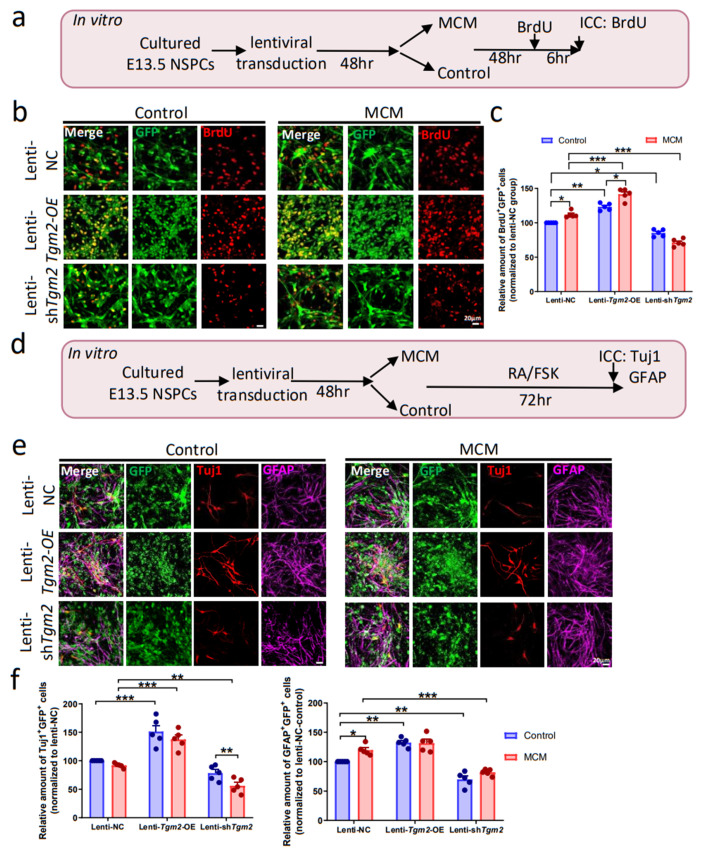
TGM2 promotes neurogenesis of NSPCs in in vitro. (**a**) Schematic illustration for analyzing proliferation of NSPCs in vitro. Cultured E13.5 NSPCs were transduced by lenti-NC, lenti-*Tgm2*-OE, or lenti-sh*Tgm2* for 48 h, then treated with control medium or microglial conditioned medium (MCM) for 48 h, followed by BrdU labeling. The ratio of BrdU^+^GFP^+^/GFP^+^ cells was quantified. (**b**,**c**) Representative images (**b**) and quantification (**c**) of BrdU immunostaining of lenti-NC, lenti-*Tgm2*-OE or lenti-sh*Tgm2*-transduced NSPCs. Scale bars: 20 µm. (**d**) Schematic illustration for analyzing differentiation of NSPCs in vitro. Cultured E13.5 NSPCs were transduced by lenti-NC, lenti-*Tgm2*-OE, or lenti-sh*Tgm2* for 48 h, then switched to differentiation medium treated with control medium or MCM for 48 h. The ratio of Tuj1^+^GFP^+^/GFP^+^ and GFAP^+^ GFP^+^/GFP^+^ cells were quantified. (**e**,**f**) Representative images (**e**) and quantification (**f**) of Tuj1 and GFAP immunostaining of the differentiation of NSPCs which were transduced with lenti-NC, lenti-*Tgm2*-OE or lenti-sh*Tgm2* virus. Scale bars: 20 µm. *n* = 5 cultures. Data are represented as means ± SEM; two-way ANOVA, * *p* < 0.05, ** *p* < 0.01, *** *p* < 0.001.

Next, we applied LPS-induced microglial conditioned medium (MCM) to characterize the role of TGM2 in regulating NSPCs proliferation under pro-inflammatory conditions in vitro. Cultured E13.5 NSPCs were transfected with lenti-NC (control), lenti-*Tgm2*-OE, or lenti-sh*Tgm2* for 48 h, then treated with MCM or control medium for 48 h, followed by BrdU labeling for 6 h (Figure 3a). As we expected, the highest amount of BrdU^+^GFP^+^ cells was observed in NSPCs treated with both lenti-*Tgm2*-OE and MCM (*F*_(2,24)_ = 25.30, *p* < 0.001). Indeed, both TGM2 overexpression (*F*_(2,24)_ = 255.8, *p* < 0.001) and MCM treatments (*F*_(1,24)_ = 7.493, *p* < 0.05) enhanced the proliferation of NSPCs. In contrast, compared with lenti-NC group, *Tgm2* knockdown led to a decreased proliferation ability of NSPCs with either control medium or MCM (Figure 3b,c). To further confirm the role of TGM2 in NSPCs proliferation, we performed neurosphere assays and found that TGM2 overexpression in NSPCs caused bigger neurospheres, while TGM2 knockdown resulted in smaller neurospheres, proving that TGM2 promotes self-renewal and proliferation of NSPCs (Figure S3g,h).

To analyze the role of TGM2 in NSPCs differentiation in vitro, the ratios of newborn neurons (GFP^+^Tuj1^+^) and new-formed astrocytes (GFP^+^GFAP^+^) to lentivirus-transduced NSPCs (GFP^+^) were quantified under pro-inflammatory conditions in vitro (Figure 3d). Our results showed that overexpression of TGM2 promoted the neuronal differentiation of NSPCs under both pro-inflammatory and normal conditions (Figure 3e,f). Knockdown of TGM2 significantly reduces the neuronal differentiation of NSPCs with MCM treatment.

Under normal conditions of astrocytic differentiation, overexpression and knockdown of TGM2 significantly promoted and inhibited astrocytic differentiation of NSPCs, respectively (Figure 3e,f). With the treatment of MCM, overexpression of TGM2 could not further enhance the differentiation of astrocytes, while knockdown of TGM2 could significantly inhibit astrocytic differentiation of NSPCs. Similar changes in *Tuj1* and *Gfap* mRNA levels were observed in NSPCs treated with MCM or TGM2 overexpression (Figure S3e,f).

### 3.4. Overexpression of TGM2 Promotes NSPCs Migration In Vitro

The scratch-wound assay was performed to investigate whether TGM2 regulates migration of NSPCs. We found that both microglial conditioned medium (*F*_(1,18)_ = 22.87, *p* < 0.001) and TGM2 overexpression (*F*_(2,18)_ = 32.62, *p* < 0.001) promoted the migration of NSPCs (Figure S3i,j). Therefore, the longest migration was observed in NSPCs treated with both lenti-*Tgm2*-OE and microglial conditioned medium. These results suggested TGM2 promotes NSPCs migration under pro-inflammatory condition.

### 3.5. Overexpression of TGM2 Enhance Adult Neurogenesis after TBI

**Figure 4:**
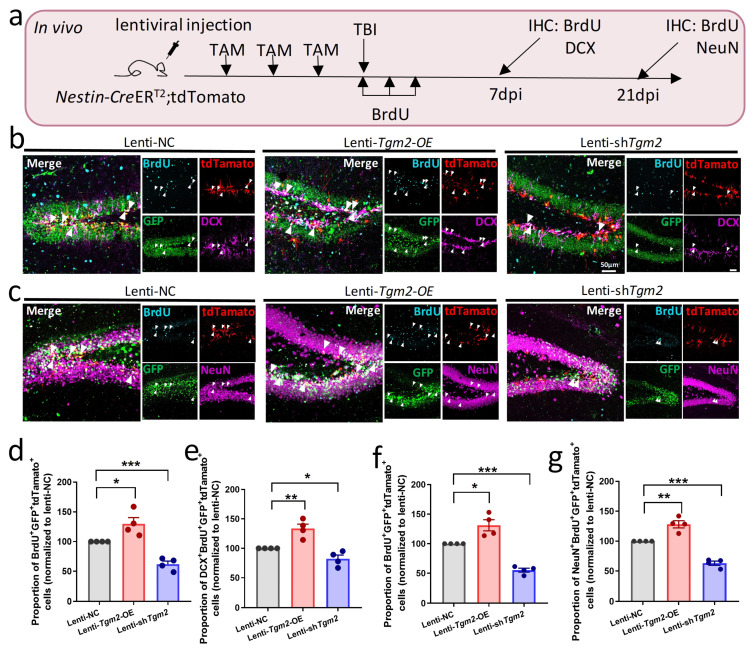
Overexpression of TGM2 promotes neurogenesis after TBI. (**a**) Schematic illustration for analyzing neurogenesis in TBI hippocampi which were injected with lenti-NC, lenti-*Tgm2*-OE, or lenti-sh*Tgm2* virus. (**b**–**g**) Representative images (**b**,**c**) and quantification (**d**–**g**) of BrdU (blue), DCX (pink) or NeuN (pink) immunostaining of hippocampal sections from *Nestin-Cre*ER^T2^;tdTomato mice which were injected with lentivirus, tamoxifen, and BrdU at given time windows. Percentages of BrdU^+^ GFP^+^ tdTamato^+^ cells among GFP^+^ tdTamato^+^ cells were quantified to examine proliferative potential of lentivirus-transduced NSPCs at 7 dpi (**c**) and 21 dpi (**f**), respectively. Percentages of BrdU^+^ GFP^+^ tdTamato^+^ DCX^+^ cells (newborn immature neurons) or BrdU^+^ GFP^+^ tdTamato^+^ NeuN^+^ cells (newborn mature neurons) among BrdU^+^ GFP^+^ tdTamato^+^ cells were quantified to determine the ability of neuronal differentiation of lentivirus-transduced NSPCs at 7 dpi (**d**) and 21 dpi (**g**), respectively. Scale bars: 50 µm. *n =* 4 mice per group. Data are represented as means ± SEM; two-tailed *t*-test, * *p* < 0.05, ** *p* < 0.01, *** *p* < 0.001.

To examine whether TGM2 regulates adult neurogenesis in vivo, we grafted lenti-NC, lenti-*Tgm2*-OE, or lenti-sh*Tgm2* into DG of 6–8 weeks old *Nestin-Cre*ER^T2^;tdTomato mice, which were then received intraperitoneal administration of TAM for three consecutive days. On the day after the last TAM injection, mice were received a stab wound injury in the hippocampus, as well as BrdU (200 mg/kg body weight) injection daily for three consecutive days (Figure 4a). At 7 days after the last BrdU injection, overexpression of TGM2 resulted in a significant increase in the proportion of BrdU^+^GFP^+^tdTomato^+^ cells among GFP^+^tdTomato^+^ cells as well as in the proportion of DCX^+^BrdU^+^GFP^+^tdTomato^+^ cells among BrdU^+^GFP^+^tdTomato^+^ cells, while knockdown of TGM2 significantly reduced the proliferation and neural differentiation of NSPCs compared to lenti-NC-transduced NSPCs (Figure 4b,d,e).

Similarly, we observed that knockdown of TGM2 significantly reduced but overexpression of TGM2 significantly enhanced the proliferation of NSPCs and increased the number of newborn neurons (BrdU^+^GFP^+^ tdTomato^+^NeuN^+^) compared to lenti-NC-transduced NSPCs at 21 days after the last BrdU injection (Figure 4c,f,g). Taken together, these results supported the idea that TGM2 has a therapeutic potential for repair after TBI by promoting endogenous neurogenesis.

### 3.6. TGM2 Regulates the Expression of Genes Associated with Proliferation and Differentiation of NSPCs

**Figure 5:**
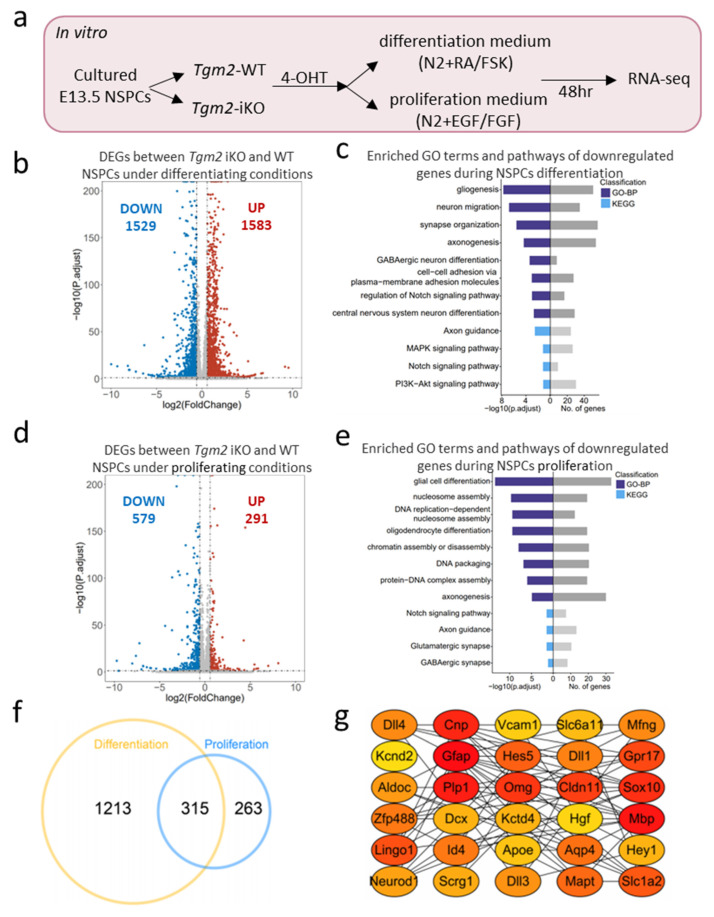
*Tgm2*-deletion alters the expression of genes related to cell proliferation and neural differentiation. (**a**) Workflow diagram for transcriptome sequencing of *Tgm2* iKO and WT NSPCs under proliferating or differentiating conditions. (**b**) Volcano plot illustrating the differentially expressed genes (DEGs) between *Tgm2* iKO and WT NSPCs under differentiating conditions. Red, upregulated genes; Blue, downregulated genes. (**c**) Bar plot depicting the significantly enriched GO terms (biological processes, BP) and KEGG pathways of downregulated genes during neural differentiation of TGM2-null NSPCs. (**d**) Volcano plot illustrating the DEGs between *Tgm2* iKO and WT NSPCs under proliferating conditions. Red, upregulated genes; blue, downregulated genes. (**e**) Bar plot depicting the significantly enriched GO terms (biological processes, BP) and KEGG pathways of downregulated genes during proliferation of TGM2-null NSPCs. (**f**) Venn plot illustrating the co-downregulated genes in processes of proliferation and differentiation. (**g**) Netplot depicting the linkages of TOP 30 co-downregulated genes as a network in processes of cell proliferation and neural differentiation.

To explore the molecular mechanism on how TGM2 regulates the proliferation and differentiation of NSPCs, we performed a transcriptional analysis of *Tgm2*-WT and *Tgm2*-iKO NSPCs that were cultured in the differentiation or proliferation medium for 48 h, respectively (Figure 5a). Results from the Pearson correlations and principal components analysis (PCA) demonstrated that there was a clear discrimination between *Tgm2*-WT and *Tgm2*-iKO NSPCs, indicating that deletion of *Tgm2* lead distinctly to gene expression pattern in both differentiating and proliferating NSPCs (Figure S4a,b).

In differentiating NSPCs, TGM2-deletion led to upregulation of 1583 genes as well as downregulation of 1529 genes (Figure 5b). To identify the biological functions perturbed by TGM2 deletion, we subjected the differentially expressed genes (DEGs) to gene ontology (GO) analysis of biological processes and Kyoto Encyclopedia of Genes and Genomes (KEGG) analysis. GO analysis showed that genes downregulated on TGM2 ablation are involved in neurogenesis and neuronal maturation including gliogenesis, synapse organization, axonogenesis, GABAergic neuron differentiation, and central nervous system neuron differentiation (Figure 5c). KEGG analysis showed that genes associated with MAPK signaling pathway, PI3K-Akt signaling pathway, and notch signaling pathway are significantly down-regulated by TGM2 deletion (Figure 5c).

In proliferating NSPCs, TGM2 deletion led to decreased expression of 579 genes as well as increased expression of 291 genes (Figure 5d). GO analysis showed that downregulated genes were associated with DNA replication-dependent nucleosome assembly, DNA packaging, and protein–DNA complex assembly, suggesting that TGM2 might mediate histone modification through transamidation [35,36,37]. KEGG analysis showed that Notch signaling pathway was significantly down-regulated in proliferating *Tgm2*-iKO NSPCs (Figure 5e).

The upregulated genes in *Tgm2*-iKO NSPCs were associated with regulation of angiogenesis, inactivation of MAPK activity, regulation of vasculature development, chemokin-mediated signaling pathway, and TNF, p53 and HIF-1 pathways (Figure S5c,d), suggesting that TGM2 might play a pivotal role in the crosstalk between NSPCs and other cell types after TBI.

As TGM2 regulated both proliferation and differentiation progress of NSPCs, we speculated that there might exist a shared molecular mechanism. Indeed, there were 315 common downregulated genes between proliferating and differentiating *Tgm2*-iKO NSPCs (Figure 5f), including Notch pathway genes (*Hes1*, *Hes5*, *Hey2*, *Dll1,* and *Dll3*), marker genes for mature astrocytes, oligodendrocytes, and neurons (*Gfap*, *Cnp*, *Mbp*, *Dcx*), neurogenesis regulation and synapse formation genes (*Sox10*, *Neurod1*, *Slc1a2*, *Apoe*, *Aqp4*), as well as regulatory genes for oligodendrocyte development and myelination (*Gpr17*, *Plp1*, *Omg*). The top 30 co-downregulated genes composed a complex regulatory network in processes of proliferation and differentiation of *Tgm2*-iKO NSPCs (Figure 5g).

**Figure 6:**
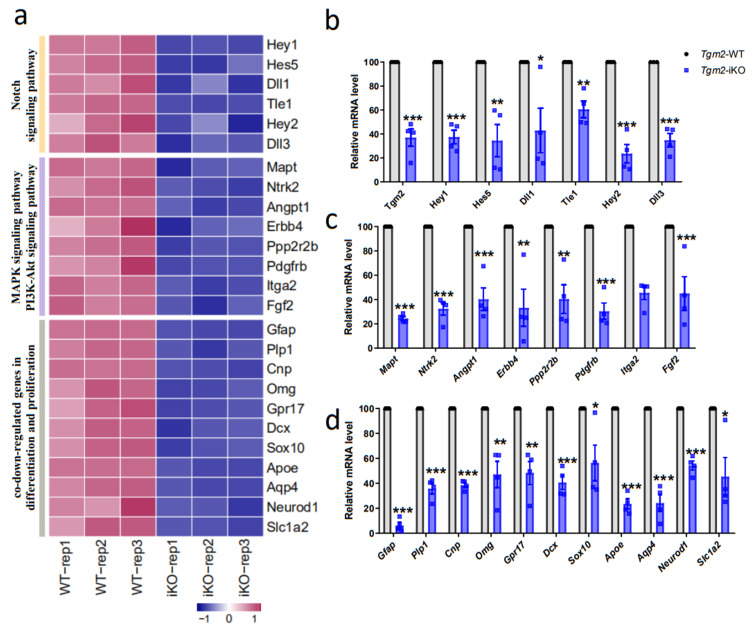
Deletion of *Tgm2* dysregulates the expression of genes associated with the proliferation and differentiation of NSPCs. (**a**) Heat map diagrams of differentially expressed Notch and MAPK signaling-pathway genes between WT and *Tgm2* iKO NSPCs in the differentiation process, as well as co-downregulated genes in processes of proliferation and differentiation of *Tgm2* iKO NSPCs. (**b**–**d**) Quantitative PCR analysis validated the downregulation of genes associated with Notch signaling (**b**), MAPK and PI3-Akt signaling pathways (**c**) and co-downregulated genes in both proliferation and differentiation processes (**d**) using a new culture of *Tgm2* iKO NSPCs under differentiating conditions. *n =* 4 cultures. Data are represented as means ± SEM; two-tailed *t*-test, * *p* < 0.05, ** *p* < 0.01, *** *p* < 0.001.

To validate these downregulated genes during proliferation and neural differentiation processes identified by RNA-seq, qRT-PCR assays were performed with RNA samples of primary NSPCs cultured with differentiation medium or proliferation medium. Indeed, the mRNA levels of *Gfap*, *Cnp*, *Dcx*, *Gpr17*, *Plp1*, *Omg*, *Sox10*, *Neurod1*, *Slc1a2*, *Apoe*, *Aqp4*, Notch pathway genes (*Hes1*, *Hes5*, *Hey2*, *Dll1* and *Dll3*), and MAPK/PI3K-Art pathway genes (*Mapt*, *Ntrk2*, *Angpt1*, *Erbb4*, *Ppp2r2b*, *Pdgfrb*, *Itga2*, and *Fgf2*) were consistently downregulated during neural differentiation of newly collected *Tgm2*-iKO NSPCs (Figure 6a-d). Moreover, downregulation of *Plp1*, *Cnp*, *Omg*, *Gpr17*, *Dcx*, *Apoe*, *Aqp4*, *Slc1a2,* and Notch pathway genes (*Hes1*, *Hes5*, *Dll1*, *Tle1,* and *Dll3*) was validated in newly collected *Tgm2*-iKO NSPCs that were cultured in proliferation medium (Figure S5a–c).

## 4. Discussion

TBI is a serious global health problem with deficiencies in prevention, care, and research [1]. The hippocampus plays a critical role in cognitive and behavioral functions and is highly susceptible to damage after TBI [38]. Since neuronal death is often observed in the hippocampus of TBI patients [39,40], the enhancement of endogenous hippocampal neurogenesis has been considered as a potential therapeutic approach for neuronal repair in the damaged hippocampus [41,42,43]. Immediately after TBI, microglia are the primary innate immune cells of the brain that rapidly respond within minutes and secrete inflammatory cytokines, neurotransmitters, chemokines, and reactive oxygen species, which produce either detrimental or favorable conditions for neurogenesis [6,44,45,46]. Lipopolysaccharide (LPS), an endotoxin from Gram-negative bacteria, can induce robust microglial inflammatory activation. The addition of conditioned medium derived from LPS-stimulated microglia to cultured NSPCs has been widely used to study the inflammatory blockade in the process of neurogenesis [47,48]. Using both a mouse model of hippocampal stab wound injury and an in vitro cell culture model, this study elucidates a novel role of TGM2 in regulating hippocampal neurogenesis following TBI. We find that deletion or knockdown of TGM2 depletes proliferating NSPCs and inhibits neuronal differentiation in the hippocampus under both physiological and TBI conditions. In contrast, overexpression of TGM2 by lentiviral transduction promotes NSPCs migration and promotes hippocampal neurogenesis after TBI. Our data suggest that TGM2 may be a therapeutic target for the enhancement of endogenous neurogenesis in the injured hippocampus.

TGM2 is widely expressed in a variety of cell types. In A431 tumor cells, TGM2 activates PI3K/Akt signaling pathway that results in the upregulation of MMP-9 and an increase in cell adhesion, migration, invasion, and cancer metastasis [49]. TGM2 overexpression triggers stem cell differentiation via PI3K/Akt signaling in glioma [50]. We find that dozens of genes associated with PI3K-Akt signaling pathway, MAPK signaling pathway, and Notch signaling pathway are significantly down-regulated in NSPCs with TGM2-deletion, suggesting these dysregulated genes may be direct or indirect downstream targets of TGMS in regulation hippocampal neurogenesis. For example, the transcription factor SP1 can be crosslinked by TGM2, which results in Sp1 inactivation [51,52,53]. Moreover, *Sp1* knockdown promotes the activation of Notch signaling pathway [54]. We speculate that TGM2 may regulate Notch signaling pathway through suppressing SP1.TGM2 has a multifunctional enzyme activity of isopeptidase, GTPase, and ATPase, deamidation, crosslinking, as well as adapter/scaffold activity [55]. TGM2 plays a prosurvival role in injured liver [56] as well as in ischemic stroke [57,58], while TGM2 accumulation results in cell death in hepatocytes [59], neuroglastoma cells [60], and neurons [61]. Although our data support that TGM2 has a prosurvival role in NSPCs by promoting their proliferation and neuronal differentiation, future studies are needed to determine the domains, conformations, and intracellular localizations of TGM2 that its function relies upon.

TBI is composed of both primary and secondary injuries that cause a series of structural damage and functional deficits in the brain [62]. Although various animal models of TBI have been developed, none of them can fully recapitulate all the pathophysiological aspects of human TBI patients [5,63]. Our previous study demonstrates that unilateral hippocampal blade stab injury (HBSI) can capture hemorrhage, neuroinflammation, and neuronal apoptosis in the injured hippocampus, but HBSI belongs to mainly focal, not diffuse type of injury [30]. In the diffuse type of TBI (such as controlled cortical impact, CCI), most newly generated neurons do not survive, and only a few of them connect with neural circuits [64,65]. Moreover, some newborn neurons are reported to aberrantly migrate in the outer granular cell layer of the hippocampus in adult mice 48 h after a moderate CCI [66]. Given that diffuse TBI is the most common type of brain injuries and tissue TGM2 has been identified as a key regulator for histone glutamine modifications, either serotonylation in the context of cellular differentiation or dopaminylation in the dopaminergic reward pathway [35,67], it will be interesting and important to investigate whether and how tissue and intracellular TGM2 play essential roles in every kinds of brain cells in various TBI models before translating findings from animals to human patients with neurotrauma.

Many specific inhibitors have been developed against TGM2 [68]. For example, ERW1041E is a TGM2-specific irreversible inhibitor that has been used to explore the role of TGM2 in hypoxia-induced pulmonary, celiac disease and cardiac fibrosis [69,70,71]. TGM2 inhibitors should be very useful to further support the role of TGM2 in cell cultures. Given that TGM2 is ubiquitously expressed in the central nervous system, technique for allowing the specific delivery of TGM2 inhibitors to NSPCS in the brain still remains a challenge. Moreover, additional experiments are required to ascertain the role and mechanisms of TGM2 in the pathogenesis of TBI, such as blocking TGM2 with specific inhibitors, profiling of proteome, rescuing TGM2-knockout phenotype, as well as evaluating possible crosstalk between different sources of TGM2.

In conclusion, the present study provides evidence showing that TGM2 is a key regulator for promoting hippocampal neurogenesis under both physiological and TBI conditions. We have also proposed some signaling pathways and gene candidates that are worthy to further explore the mechanisms of altered neurogenesis and to develop cell-based therapeutic strategies for treating brain trauma as well as other neurodegenerative diseases. Hence, this study fills the gap of TGM2 in adult neurogenesis and proves the feasibility of endogenous NSPCs-based therapeutics for brain repair.

## Figures and Tables

**Table 1:** The primers for qRT-PCR.

Gene		Sequence (5′–3′)
*Angpt1*	forward	CACATAGGGTGCAGCAACCA
	reverse	CGTCGTGTTCTGGAAGAATGA
*Apoe*	forward	CTGACAGGATGCCTAGCCG
	reverse	CGCAGGTAATCCCAGAAGC
*Aqp4*	forward	CTTTCTGGAAGGCAGTCTCAG
	reverse	CCACACCGAGCAAAACAAAGAT
*Cnp*	forward	ACGAGTGCAAGACGCTATTCA
	reverse	GGTGCCGTCGTGGTACTTC
*Dcx*	forward	CATTTTGACGAACGAGACAAAGC
	reverse	TGGAAGTCCATTCATCCGTGA
*Dll1*	forward	CAGGACCTTCTTTCGCGTATG
	reverse	AAGGGGAATCGGATGGGGTT
*Dll3*	forward	CTGGTGTCTTCGAGCTACAAAT
	reverse	TGCTCCGTATAGACCGGGAC
*Erbb4*	forward	TCCCCCAGGCTTTCAACATAC
	reverse	GCTGTGTCCAATTTCACTCCTA
*Fgf2*	forward	GCGACCCACACGTCAAACTA
	reverse	TCCCTTGATAGACACAACTCCTC
*Gfap*	forward	CCCTGGCTCGTGTGGATTT
	reverse	GACCGATACCACTCCTCTGTC
*Gpr17*	forward	CACCCTGTCAAGTCCCTCAAG
	reverse	GTGGGCTGACTAGCAGTGG
*Hey1*	forward	GCGCGGACGAGAATGGAAA
	reverse	TCAGGTGATCCACAGTCATCTG
*Hey2*	forward	AAGCGCCCTTGTGAGGAAAC
	reverse	GGTAGTTGTCGGTGAATTGGAC
*Hes5*	forward	AGTCCCAAGGAGAAAAACCGA
	reverse	GCTGTGTTTCAGGTAGCTGAC
*Itga2*	forward	TGTCTGGCGTATAATGTTGGC
	reverse	CTTGTGGGTTCGTAAGCTGCT
*Mapt*	forward	CGCTGGGCATGTGACTCAA
	reverse	TTTCTTCTCGTCATTTCCTGTCC
*Neurod1*	forward	ATGACCAAATCATACAGCGAGAG
	reverse	TCTGCCTCGTGTTCCTCGT
*Ntrk2*	forward	CTGGGGCTTATGCCTGCTG
	reverse	AGGCTCAGTACACCAAATCCTA
*Omf*	forward	CTTCCTGCCTGTTCATCCTTC
	reverse	ATCCAGGGTTCTCAGATTGGT
*Plp1*	forward	CCAGAATGTATGGTGTTCTCCC
	reverse	GGCCCATGAGTTTAAGGACG
*Ppp2R2B*	forward	ACGGGAGAGTTACTAGCGAC
	reverse	GTAAGCTGCGTTTTGTTGAGG
*Pdgfrb*	forward	TTCCAGGAGTGATACCAGCTT
	reverse	AGGGGGCGTGATGACTAGG
*Slcla2*	forward	ACAATATGCCCAAGCAGGTAGA
	reverse	CTTTGGCTCATCGGAGCTGA
*Sox10*	forward	CGGACGATGACAAGTTCCCC
	reverse	GTGAGGGTACTGGTCGGCT
*Tgm2*	forward	CGCAACAGGGCTTCATCTAC
	reverse	CCCGACTACGGTTCTTCAGGA
*Tle1*	forward	CCAGTACCTCTCACGCCTCA
	reverse	GCCCACTCAGAGCACTAGAC

## Data Availability

All datasets supporting the conclusions are included in the article. Further enquiries on data and materials can be directed to the corresponding author.

## Supplementary Materials

The following supporting information can be downloaded at: https://www.mdpi.com/article/10.3390/cells12040558/s1. Figure S1: Tamoxifen-induced conditional knockout of *Tgm2* in hippocampal NSPCs. Figure S2: TGM2 is required for hippocampal neurogenesis. Figure S3: TGM2 promotes self-renewal and migration of NSPCs. Figure S4: *Tgm2*-deletion resulted in transcriptional changes in NSPCs. Figure S5: Deletion of *Tgm2* dysregulates the expression of genes associated with NSPCs proliferation and differentiation.
