# Supplementary material for: The Role and Mechanism of Transglutaminase 2 in Regulating Hippocampal Neurogenesis after Traumatic Brain Injury
Source: Cells. 2023 Feb 9;12(4):558. doi: 10.3390/cells12040558 (PMC9954100; doi:10.3390/cells12040558)
Supplement: Supplementary file 1 [file cells-12-00558-s001.zip › cells-2094621-supplementary.pdf]

Supplemental information

Supplemental figures

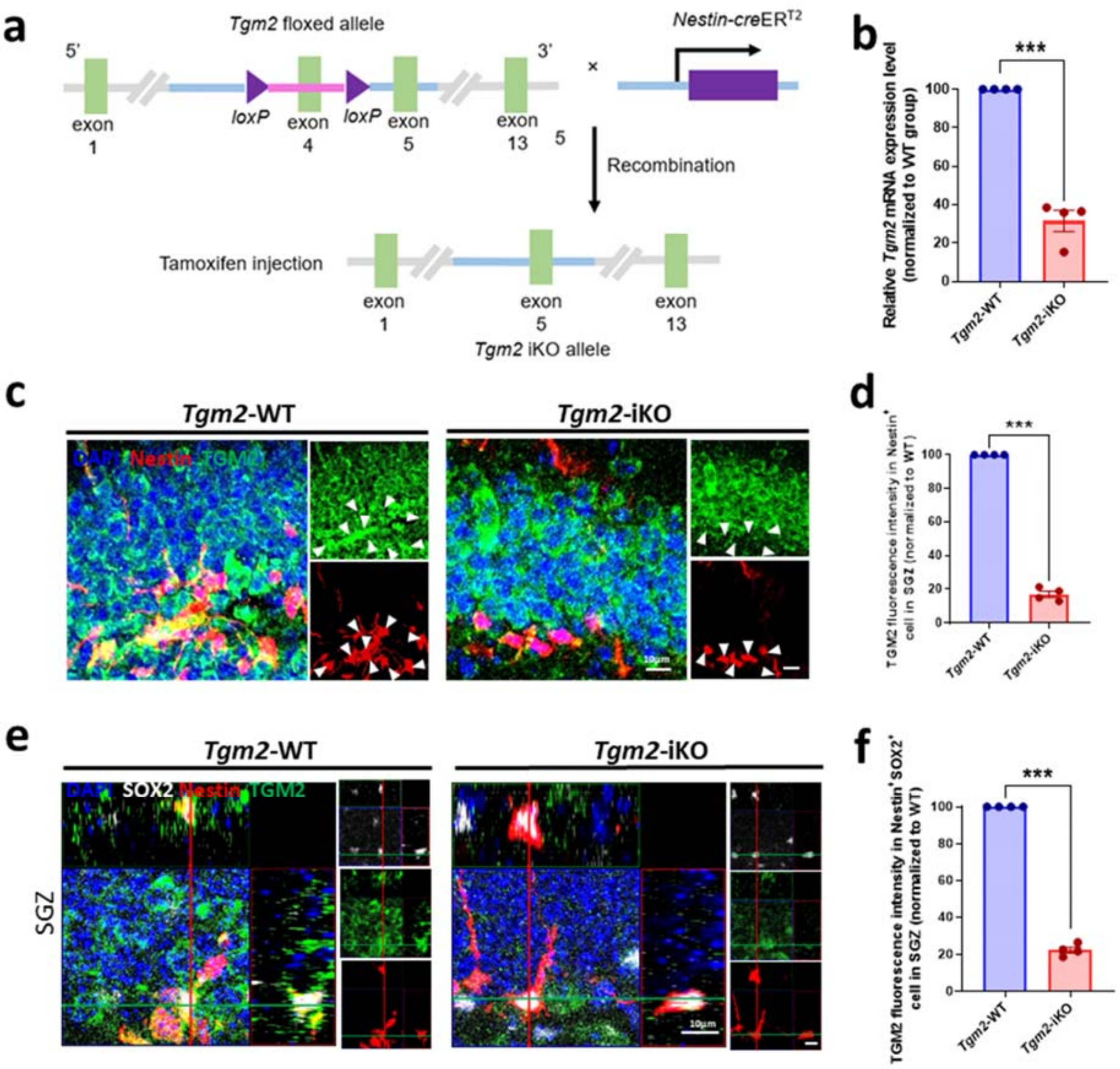

Figure S1 Tamoxifen-induced conditional knockout of *Tgm2* in hippocampal NSPCs. **(a)** Schematic diagram for the generation of NSPCs-specific tamoxifen-inducible *Tgm2* knockout (iKO) mice. **b** *Tgm2* mRNA expression levels in the dentate gyrus of *Tgm2* iKO and WT littermates by real-time qPCR. **c-d** Representative images (**c**) and quantification (**d**) of TGM2 immunostaining in NSPCs (Nestin^{+}) in the dentate gyrus of *Tgm2* iKO and WT littermates. Arrowheads indicate Nestin^{+} NSPCs. **e-f** Three-dimensional images (**e**) and quantification (**f**) of TGM2 immunostaining in

NSPCs (Nestin⁺Sox2⁺) of *Tgm2* iKO and WT littermates. Scale bars: 10 μm. $n = 4$ mice per group. Data are shown as means ± SEM; two-tailed *t*-test, ***$p < 0.001$.

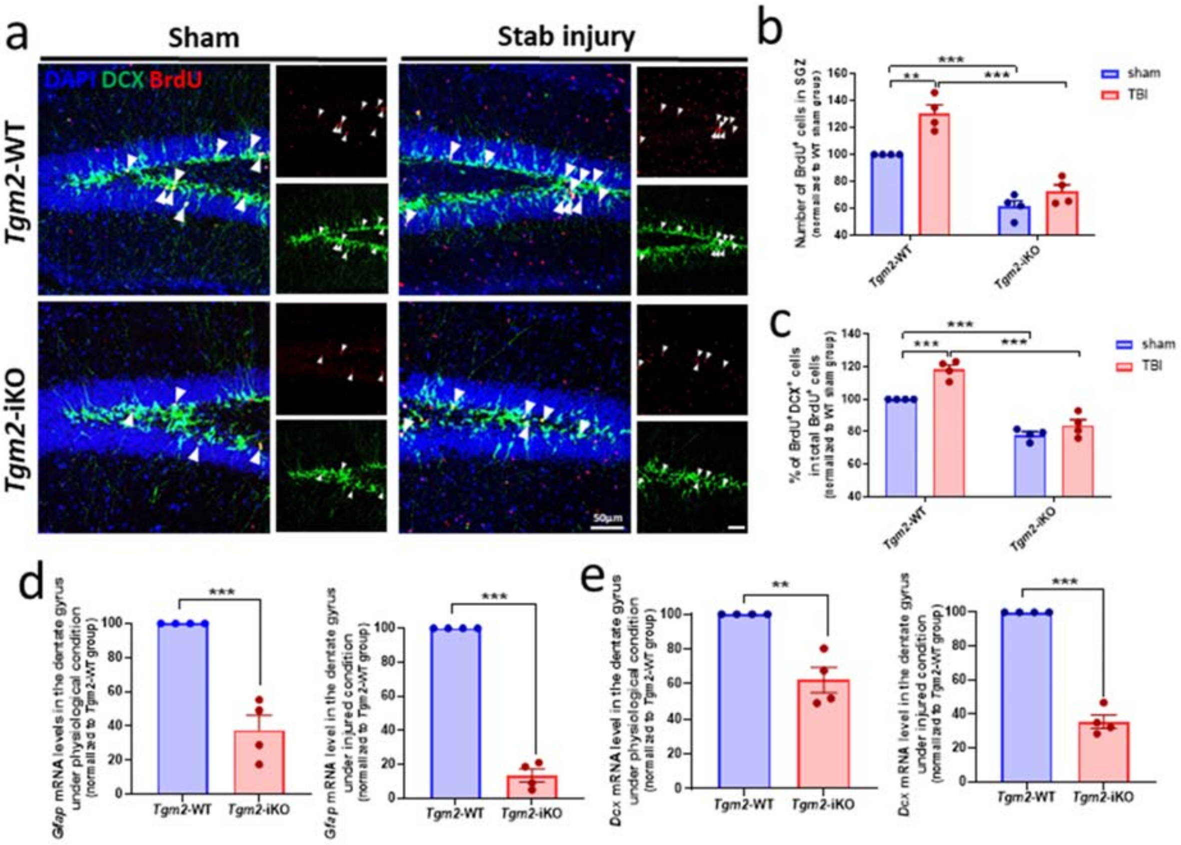

Figure S2 TGM2 is required for hippocampal neurogenesis. **(a)** Representative images of BrdU and DCX immunostainings of dentate gyrus of *Tgm2*-WT and iKO mice at 14 days after TBI and BrdU injection. Scale bars: 50 μm. **b-c** Quantification of the amounts of BrdU⁺ cells (**b**) and BrdU⁺DCX⁺ cells (**c**) (n=4). **d-e** The relative expression levels of *Gfap* (**d**) and *Dcx* (**e**) mRNA in the dentate gyrus of *Tgm2* iKO mice under both physiological and injured conditions compared to that of WT littermates by qRT-PCR. Data are represented as means ± SEM; *$p < 0.05$, **$p < 0.01$, ***$p < 0.001$.

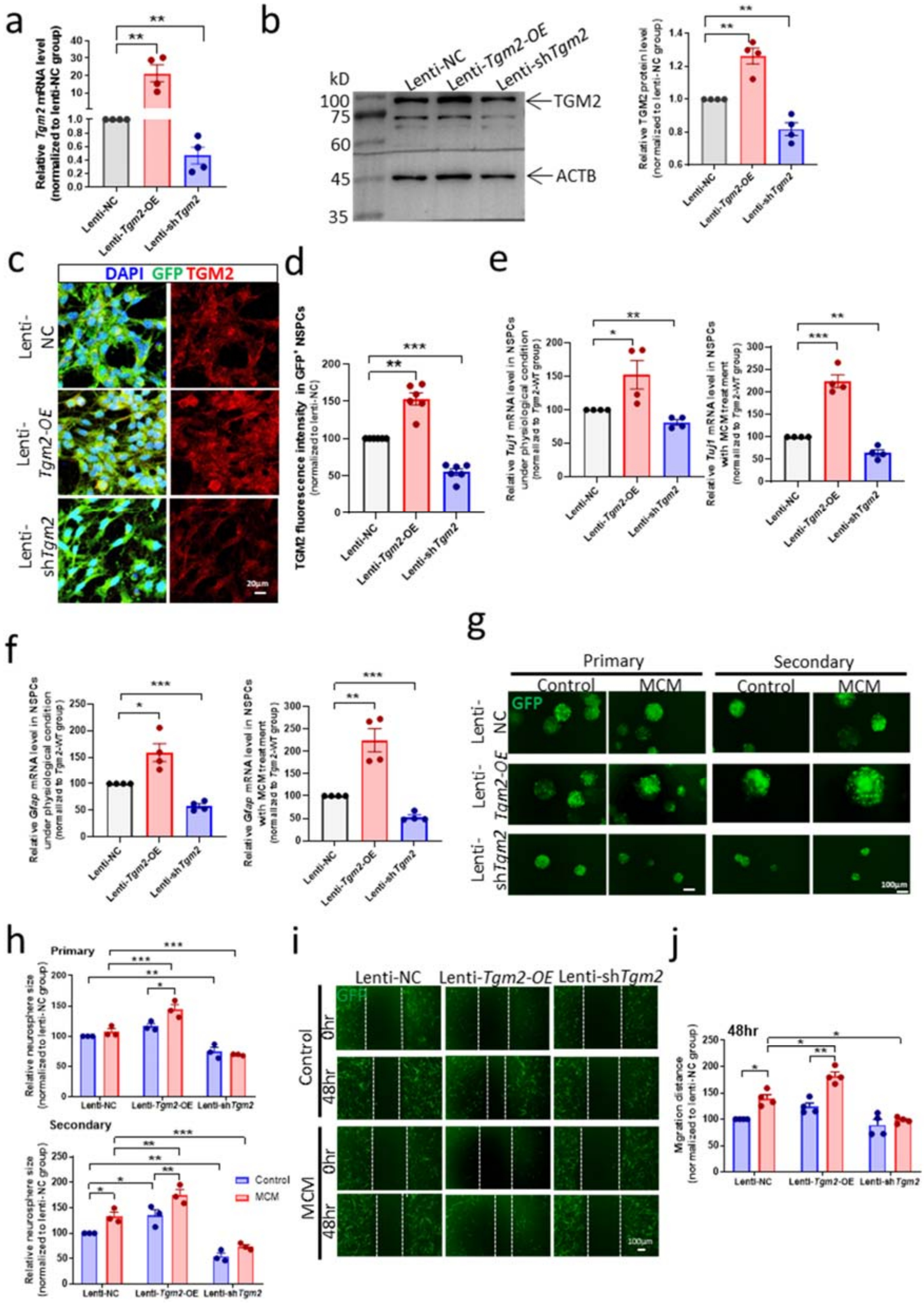
a
b
c
d
e
f
g
h
i
j
Lenti-NC
Lenti-Tgm2-OE
Lenti-shTgm2
TGM2
ACTB
kD
DAPI GFP TGM2
Primary
Secondary
Control
MCM
GFP
48hr
0hr
Migration distance (normalized to lenti-NC group)
Relative neurosphere size (normalized to lenti-NC group)

Figure S3 TGM2 promotes self-renewal and migration of NSPCs. **(a-b)** *Tgm2* mRNA (**a**) and protein (**b**) expression levels in cultured NSPCs which were transduced with lenti-NC, lenti-*Tgm2*-OE or lenti-sh*Tgm2* virus for 72h. **c-d** Representative images (**c**) and quantification (**d**) of TGM2 immunostaining in cultured NSPCs which were transduced with lenti-NC, lenti-*Tgm2*-OE or lenti-sh*Tgm2* for 72h. Scale bars: 20 μm. **e-f** The mRNA expression levels of *Tuj1* (**e**) and *Gfap* (**f**) in cultured NSPCs which were transduced with lenti-NC, lenti-*Tgm2*-OE or lenti-sh*Tgm2* for 72h. n = 4 cultures. **g-h** Representative images (**g**) and quantification (**h**) of the sizes of primary, secondary, and tertiary neurospheres formed 4 days after initial plating of NSPCs which were transduced with lenti-NC, lenti-*Tgm2*-OE or lenti-sh*Tgm2*. Scale bars: 100 μm. **i-j** Representative images (**i**) and quantification (**j**) of the migration of NSPCs in cell scratch experiment. Scale bars: 100 μm. n = 4 cultures. Data are represented as means ± SEM; two-way ANOVA, *$p < 0.05$, **$p < 0.01$, ***$p < 0.001$.

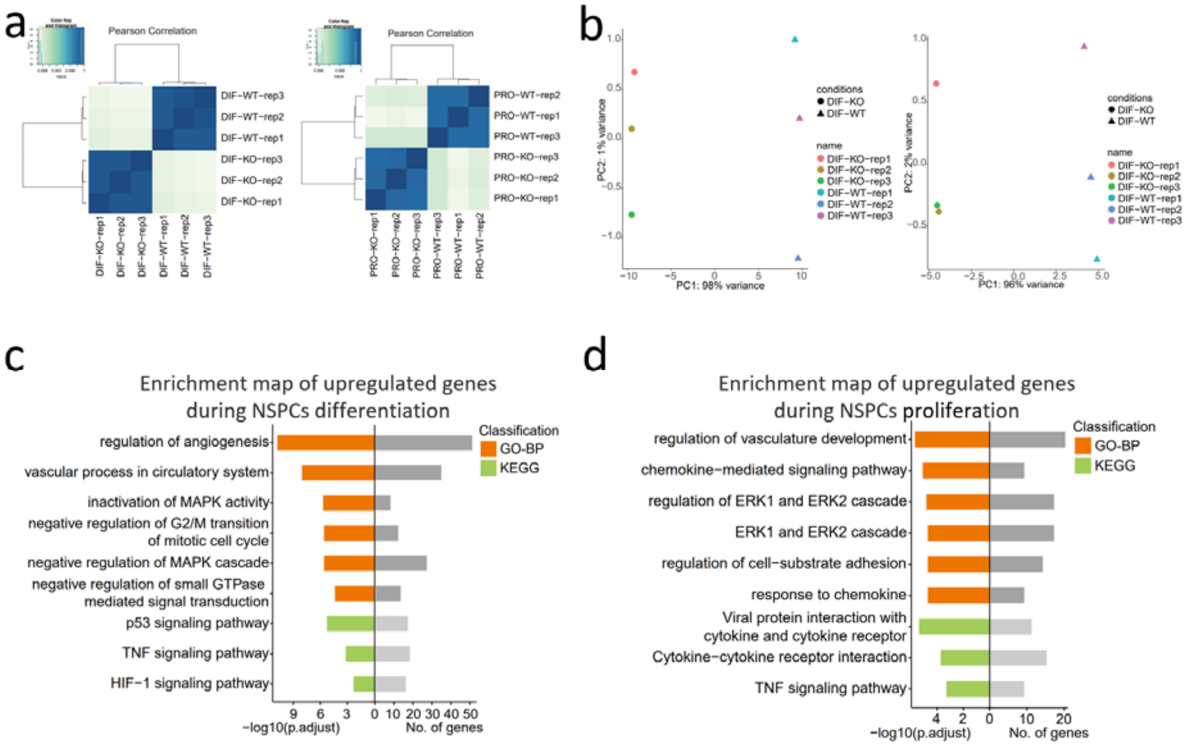

Figure S4 *Tgm2*-deletion resulted in transcriptional changes in NSPCs. **(a)** The Pearson correlations depicting the correlation coefficients of RNA-seq samples. **b** Principal component analysis (PCA) showing the variations in NSPCs transcriptomes between *Tgm2* WT and iKO groups. **c** Bar plot depicting the significantly enriched GO terms (biological processes, BP) and KEGG pathways of upregulated genes during neural differentiation of TGM2-null NSPCs. **d** Bar plot depicting the significantly enriched GO terms (biological processes, BP) and KEGG pathways of upregulated genes during proliferation of TGM2-null NSPCs.

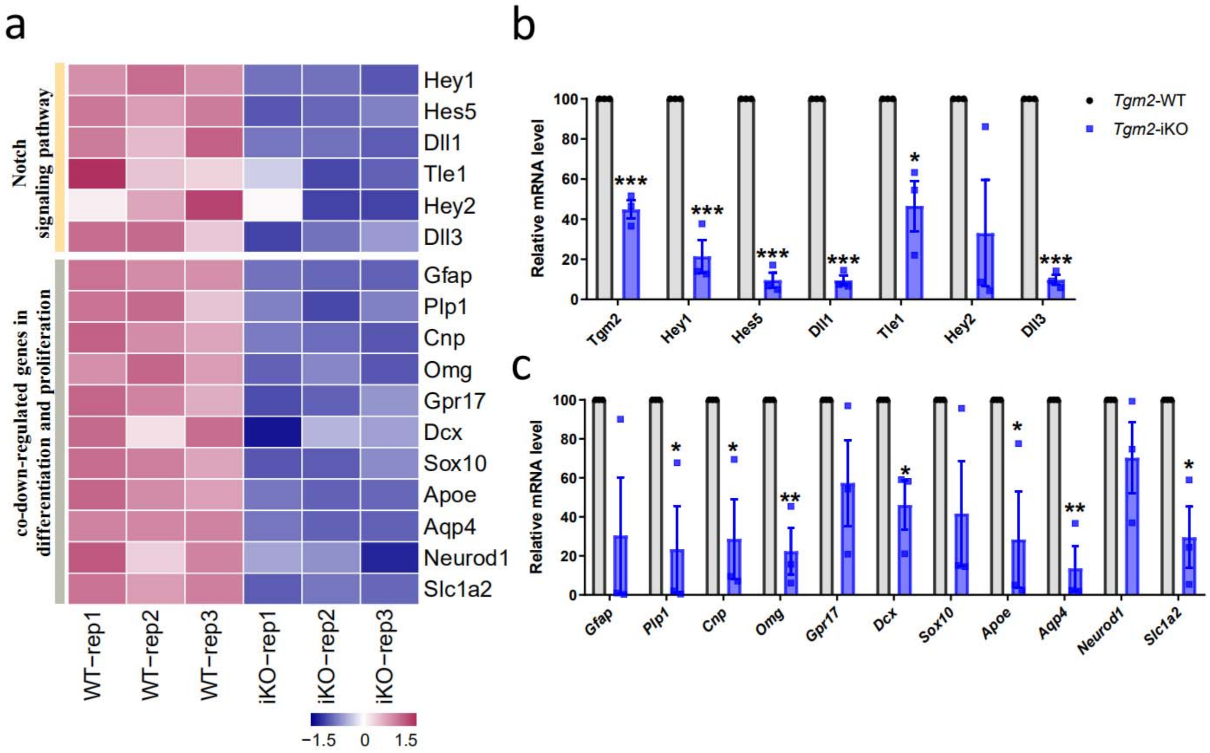

Figure S5 Deletion of *Tgm2* dysregulates the expression of genes associated with NSPCs proliferation and differentiation. **(a)** Heat map diagrams of co-downregulated genes in processes of proliferation and differentiation as well as differentially expressed Notch-signaling-pathway genes between *Tgm2* WT and iKO NSPCs in the proliferation process. **b,c** Quantitative PCR analysis verified that almost all of previously identified Notch-signaling-pathway genes **(b)** and co-downregulated genes in processes of proliferation and differentiation **(c)** were significantly downregulated in a new culture of *Tgm2* iKO NSPCs under proliferating conditions. $n = 3$ cultures. Data are shown as means ± SEM; two-tailed *t*-test, $*p < 0.05$, $**p < 0.01$, $***p < 0.001$.
